# Mitochondrial DNA variation of the ruffed grouse (*Bonasa umbellus*)

**DOI:** 10.1186/s13104-019-4607-3

**Published:** 2019-09-11

**Authors:** Rodney L. Honeycutt, Glenn A. Proudfoot, Nova J. Silvy

**Affiliations:** 1 Natural Science Division, Pepperdine University, 24255 Pacific Coast Highway, Malibu, CA 90263 USA; 2 Department of Wildlife and Fisheries Sciences, Texas A&M University, 2258 TAMU, College Station, TX 77843-2258 USA

**Keywords:** Ruffed grouse, *Bonasa umbellus*, Phylogeography, mtDNA, Historical demography

## Abstract

**Objective:**

The ruffed grouse, *Bonasa umbellus*, is broadly distributed across North America and displays considerable taxonomic diversity. Except for a genetic study of some western populations of ruffed grouse, nothing is known about genetic variation in other regions of Canada and the United States. Our objective is to examine patterns of mitochondrial DNA (mtDNA) variation in the ruffed grouse across western, central, and eastern parts of its distribution. We compare patterns of mtDNA variation to those characterized by morphology and ecology. Additionally, we evaluate the demographic history of the species based on mitochondrial haplotype diversity.

**Results:**

Patterns of mtDNA variation revealed geographic subdivision, with populations of ruffed grouse subdivided into 3 to 4 genetically distinct groups. This subdivision partially coincided with the ranges of described subspecies. Behavioral traits prohibiting long-distance movement and barriers to dispersal in response to physiography and unsuitable habitat help explain these patterns of subdivision. Historically, the ruffed grouse probably experienced a population expansion, possibly in response to changes during the Pleistocene.

## Introduction

The ruffed grouse (*Bonasa umbellus*) is the most broadly distributed species of grouse in North America, occupying early-succession deciduous and coniferous forests in the United States and Canada [1, 2]. Given its importance as a game species, the ruffed grouse has received considerable attention from those interested in its ecology and management [3]. Throughout its range, the ruffed grouse shows considerable variation of feathering on the tarsus, plumage color, and ecology [4, 5]. These variable traits contribute to the taxonomic designation of 11 [6] to 16 [7] subspecies. Although previous studies clearly document morphological and ecological differences across the ruffed grouse’s range, morphology-based taxonomy is not always congruent with geographic patterns of genetic variation [8], thus making it difficult to identify units of conservation [9]. To the best of our knowledge, the only genetic study of ruffed grouse assesses the landscape genetics of populations from the extreme western range of the species [10].

In this study, we used nucleotide sequence data from a fragment of the mitochondrial cytochrome *b* gene to: (1) determine whether patterns of genetic variation are congruent with current taxonomic designations of several subspecies and (2) test hypotheses related to the demographic history of the species.

## Main text

### Methods

#### Samples collected

Tissue biopsies (brain, heart, and liver) were collected earlier by wildlife game agents during hunting season. We examined 100 individuals, representing seven subspecies as follows: United States: *B. u. yukonesis* (Alaska AK, n = 1), *B. u. sabini* (Washington WA, n = 8), *B. u. incana* (Idaho ID, n = 2; Montana MT, n = 1; North Dakota ND, n = 8; South Dakota SD, n = 3), *B. u. monticola* (North Carolina NC, n = 3; Pennsylvania PN, n = 5; Tennessee TN, n = 2; Kentucky KY, n = 2), *B. u. togata* (Wisconsin WI, n = 21; Minnesota MN, n = 10; Vermont VT, n = 5). Canada: *B. u. umbelloides* (Alberta ALB, n = 3; Manitoba MTB, n = 14), *B. u. togata* (Quebec QUE, n = 3; Ontario ONT, n = 2; Nova Scotia NS, n = 6), *B. u. labradorensis* (Newfoundland NF, n = 1).

#### DNA extraction and PCR amplification

DNA was extracted with a DNeasy Blood and Tissue Kit (Qiagen, Valencia, California, USA). A 515 bp fragment of the mitochondrial cytochrome *b* gene was amplified using the polymerase chain reaction (PCR) with primers H15295 [11] and L14841 [12]. PCR was performed in 50 μl reactions containing: 2 μl of 10 mM solution of each primer, 5 μl of 10× buffer solution with 20 mM MgCl_2_, 4 μl dNTP mix, 0.2 μl of Takara *Ex Taq* polymerase, and 1–2 μl of DNA template. PCR reaction conditions included: (1) preliminary denaturation 1 cycle, 4 min at 94 °C; (2) 35 cycles with denaturation (1 min, 94 °C), annealing (1 min, 50 °C), extension (1 min, 72 °C; (3) 4 min extension at 72 °C.

#### Sequencing

Polymerase chain reaction products were purified with a QIAquick PCR Purification Kit (Qiagen), and sequence reactions were performed using a Big Dye Terminator Cycle sequencing kit v1.1 (Applied Biosystems, Foster City, California, USA) and an ABI 377 automated sequencer. Excess dye was removed using a Sephadex G-50 spin column (Sigma-Aldrich, St. Louis, Missouri, USA). Both strands were sequenced, and contigs were created using Sequencher 4.0 (Gene Codes Corporation, Ann Arbor, Michigan, USA).

#### Analyses

DnaSP v6 [13] was used to estimate the number of haplotypes (*h*), the average number of substitutions per site, and Nei’s [14] estimates of both haplotype diversity (*Hd*) and nucleotide diversity (π). A mitochondrial haplotype network was constructed using the TCS 1.21 [15].

Pairwise estimates of nucleotide differences between populations, estimated in DnaSP, were used in a neighbor-joining analysis in PAUP* v 4.0b10 [16]. The hazel grouse, *Bonasa bonasia* (GenBank AF230165), was used as an outgroup [17]. An analysis of molecular variance (AMOVA) in Arlequin v3.5 [18] was used to test for genetic structure among various groupings.

Several methods were used to test for historical changes in demography. We used the distribution of pairwise nucleotide differences or mismatch distribution to determine whether populations have remained constant or experienced demographic expansion [19]. The degree to which the distribution fit a constant versus expanding historical population was evaluated with the raggedness index *r* [20]. Confidence limits for how well our data fit expectations were determined using coalescent simulations (10,000 replications) in DnaSP. The hypothesis of a population expansion was evaluated using the sum of square deviations (SSD) and raggedness index in Arlequin, with confidence intervals derived from 10,000 parametric bootstraps. Fu’s *F*_*s*_ [21] was used to test for deviations from a model of constant population size, expected under a neutral mutation model.

### Results

We identified 19 mitochondrial haplotypes with an average number of nucleotide differences of 2.845. Overall average nucleotide diversity (π) = 0.00552 and haplotype diversity (*Hd*) = 0.862. Five haplotypes (1, 2, 6, 11, 14) had the highest frequency (79%), with haplotype 2 occurring in 24% of individuals followed by 17% for haplotypes 11 and 14, 15% for haplotype 1, and 5% for haplotype 6 (Table 1). Twelve haplotypes occurred in a single individual. Both the Washington and Wisconsin localities had unique haplotypes occurring at a frequency of 50% and 28%, respectively (Table 1).

**Table 1:** Percentages of mitochondrial haplotypes partitioned by state and province

Hap^a^	WA	MT	ID	AK	ALB	MTB	MN	ND	SD	ONT
1				100	66	29	40	12.5	100	100
2					34	50	30	75		
3						7				
4						7				
5						7				
6	37.5	100	50							
7	50									
8	12.5									
9			50							
10								12.5		
11							10			
12							10			
13							10			
14										
15										
16										
17										
18										
19										
*Hd* ^b^	0.68		1.00		0.67	0.70	0.80	0.46		
π^c^	0.005		0.002		0.001	0.002	0.003	0.001		

Hap^a^	WI	KY	TN	NC	QUE	NS	PN	VT	NF
1									
2	24								
3									
4									
5									
6									
7									
8									
9									
10									
11	38						80	60	
12									
13									
14	5	100	50	66	100	100	20	40	
15	28								
16	5								
17			50						
18				34					
19									100
*Hd* ^b^	0.75		1.00	0.68	0.67		0.400	0.60	
π^c^	0.003		0.01	0.002	0.0051		0.0031	0.005	

*WA* Washington, *MT* Montana, *ID* Idaho, *AK* Alaska, *ALB* Alberta, *MTB* Manitoba, *MN* Minnesota, *ND* North Dakota, *SD* South Dakota, *ONT* Ontario, *WI* Wisconsin, *KY* Kentucky, *TN* Tennessee, *NC* North Carolina, *QUE* Quebec, *NS* Nova Scotia, *PN* Pennsylvania, *VT* Vermont, *NF* Newfoundland

^a^Haplotypes

^b^Haplotype diversity (only shown for populations with more than one haplotype)

^c^Nucleotide diversity

**Fig. 1:**
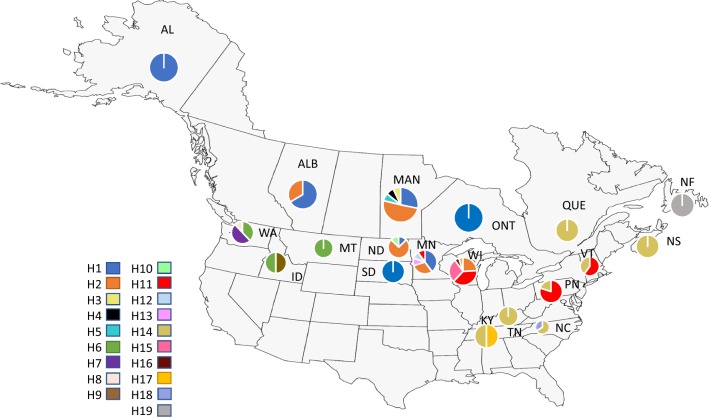
Distribution of populations sampled and frequency of haplotypes (see Table 1). This map was modified from a base map provided by Mapchart.net licensed under a Creative Commons Attribution-ShareAlike 4.0 international license. https://mapchart.net/

The frequency and distribution of haplotypes revealed a pattern suggesting geographic subdivision among localities (Table 1; Fig. 1). Haplotype 6 was restricted to WA, MT, and ID (Group 1), whereas haplotypes 1 and 2 occurred only at Group 2 localities (AK, MN, WI, ND, SD, ALB, MTB, and ONT). Haplotype 14 occurred at high frequency in Ky, TN, NC, PN, VT, and the Provinces of QUE and NS (Group 3). Haplotype 19 was unique to NF (Group 4).

Several different configurations of AMOVA were performed. First, samples were grouped by individual states and provinces to test for overall geographic variation, yielding percentage of variation among populations of 32.58% and within populations of 67.42% (F_ST_ = 0.326, *p* < 0.000). Second, an analysis of the four groups defined above resulted in an among group variation of 56.29% and 43.71% within populations (F_ST_ = 0.563, *p* < 0.000). Finally, populations within each group were compared. No significant structure among localities within Group 1 was observed (95.17% within populations, F_ST_ = 0.0483, *p* = 0.478). Significant genetic structure was observed for Group 2 (16.8% among and 83.2% within populations; F_ST_ = 0.168, *p* = 0.000) and Group 3 (46.68% among and 51.32% within populations; F_ST_ = 0.487, *p* = 0.000). The Wisconsin population accounted for the majority of among population variation in Group 2. After removal of the Wisconsin population, no evidence of genetic structure was observed for the remaining populations in Group 2 (98.21% within, *p* = 0.358). Vermont and Pennsylvania accounted for most of the among population differences in Group 3, and with their removal no genetic structure was observed (*p* = 0.227). Wisconsin, Vermont, and Pennsylvania shared haplotype 11 in high frequency, and when these three localities were combined, no significant genetic differentiation was observed (*p* = 1.668).

**Fig. 2:**
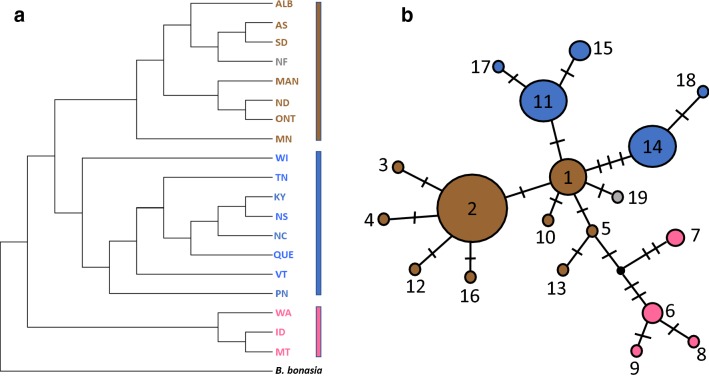
**a** Neighbor-joining phenogram based on pairwise comparisons of average nucleotide differences. Abbreviations for localities are the same as those provided in Table 1. Group 1 (pink), Group 2 (blue), Group 3 (brown), Newfoundland (Grey). **b** Haplotype network with numbers corresponding to haplotypes described in Table 1. Cross marks represent single mutations, and the dark dot represents an unknown haplotype

Populations were grouped according to individual states and provinces, and the average number of nucleotide differences between pairs of populations was used to produce a neighbor-joining tree in PAUP* (Fig. 2a), resulting in a dendrogram coinciding with the general pattern seen in Table 1. A neighbor-joining analysis based on p-distance and 10,000 bootstrap replications for the 19 haplotypes and the outgroup revealed some support for Groups 2 and 3 (see Additional file 1). This pattern can be seen in the haplotype network (Fig. 2b), which revealed a star-like arrangement with individual haplotypes separated by a small number of mutations. Division of lineages in Group 3 identified by the AMOVA are reflected in the network by the separation of haplotypes 11 and 14.

The mismatch distribution was unimodal and deviated from that expected under a neutral model or constant population size, with the raggedness index of 0.0248 (*p* > 0.05) non-significant based on DnaSP. The generalized least-square approach in Arlequin was used to test for a population expansion, resulting in non-significance with SSD *p* = 0.544 and *p* = 0.671 for the raggedness index. For the standard neutral model examined in DnaSP Fu’s *F*_*s*_= − 5.51511 (*p* = 0.035). Arlequin produced a similar value of Fu’s *F*_*s*_ (*p* = 0.038).

### Discussion

Geographic variation in ruffed grouse, as characterized by the number of recognized subspecies, is based primarily on morphological features of coloring, degree feathering on the tarsus, feathers on the toes, and ecology [4, 5]. Size of the overall ranges of recognized subspecies varies, with *B. u. brunnescens* restricted to parts of British Columbia and Vancouver Island and *B. u. umbelloides* occurring from southeastern Alaska to Quebec as well as areas in several western states [5, 7]. Validation of these subspecies requires an independent assessment of genetic variation [9].

Other studies of grouse have shown partial correspondence between morphologically named subspecies and identified genetic units [22, 23]. Although not completely congruent with currently designated subspecies of ruffed grouse, observed patterns of genetic variation confirm that ruffed grouse show evidence of population subdivision, and some of this variation coincides with portions of the range of recognized subspecies. For instance, a recent study [10] based on microsatellite and mtDNA markers revealed high levels of population differentiation of populations from Alaska and Washington, with evidence of subdivision for populations from western Canada. Our data indicate that populations containing individuals from Washington, Idaho, and Montana (Group 1) represent one of the most distinct and divergent groups of ruffed grouse. Although *B. u. sabini* is one of the subspecies occupying Washington, populations in Idaho and Montana are assigned to *B. u. umbelloides*. With only one sample from Alaska, we are unable to confirm the uniqueness of *B. u. yukonesis*, which appears unique based on a previous study [10]. *B. u. umbelloides* is one of the most widely distributed subspecies, and our Group 2 encompasses a large portion of this subspecies’ range. Group 3 is more complicated, overlapping with *B. u. togata* and *B. u. monticola*, and even with consideration of populations from Wisconsin, Pennsylvania, and Vermont as a separate group, the variation observed (Fig. 1) does not coincide with the designated ranges of these two subspecies. More recently, *B. u. labradorensis* was recognized as a separate subspecies [24], and the haplotype from our single sample was unique. Clearly, a more detailed study of this geographic region is merited.

Although Fu’s *F*_*s*_ is negative and has a *p* value < 0.05, it is still higher than the recommended significance threshold of 0.02. However, all other analyses support an historical population expansion. The haplotype network and small number of mutations separating haplotypes reveal a star-like “gene tree” that is coincident with an expansion [25], the DnaSP analysis revealed a unimodal mismatch distribution, and the test in Arlequin also supported a population expansion. Part of this discrepancy between these observations and Fu’s *F*_*s*_ may be resolved with increased sampling [26].

Diversification of grouse appears to be an early to middle Pleistocene event [27], and coalescence of gene genealogies of many species confirms the influence of changes occurring during the Pleistocene [26]. Our genetic data and the previous study on western populations reveal patterns of geographic subdivision in a species known to prefer specific plant communities [5] and to display restricted movement patterns in response to both predation [28] and restricted dispersal across unsuitable habitat [10]. The extent to which these factors help explain the expansion of ruffed grouse during the Pleistocene requires a broader survey of genetic variation across the species’ range.

## Limitations

Although populations were sampled throughout a large portion of the ruffed grouse’s range, not all subspecies were sampled, and at some localities, sample size was limited. Our genetic data are restricted to a mitochondrial marker, and inclusion of other genetic markers is likely to improve gene genealogies, thus allowing for a more detailed assessment of geographic variation in this broadly distributed species.

## Supplementary information

**Additional file 1.** Neighbor-joining Tree of Haplotypes. This is a phenogram based on p-distances and produced with a neighbor-joining analysis (10,000 bootstrap replications) that shows relationships among the 19 haplotypes.

**Additional file 2.** Nucleotide Sequences Used for Analyses. This spreadsheet contains the complete sequences of the 100 individual ruffed grouse plus the outgroup taxon used in this study. Individual subspecies are cross-referenced, and individual sequences representing the 19 haplotypes used for Table 1 and Figs. 1 and 2 are identified.

## Data Availability

All 100 ruffed grouse sequences plus the outgroup are included in a spreadsheet designated Additional file 2. In addition to the sequences, the spreadsheet identifies the 19 haplotypes presented in Table 1, Figs. 1 and 2. Any further information is available upon request.

## Abbreviations

ABI: Applied Biosystems
*B. u.*: *Bonasa umbellus*
DNASP: DNA sequence polymorphism
mtDNA: mitochondrial DNA
TCS: Estimating Gene Genealogies
PCR: polymerase chain reaction
AK: Alaska
ALB: Alberta
KY: Kentucky
ID: Idaho
MN: Minnesota
MTB: Manitoba
MT: Montana
NC: North Carolina
ND: North Dakota
NF: Newfoundland
NS: Nova Scotia
ONT: Ontario
PN: Pennsylvania
QUE: Quebec
SD: South Dakota
TN: Tennessee
VT: Vermont
WA: Washington
WI: Wisconsin
