# Supplementary material for: Mitochondrial DNA variation of the ruffed grouse (Bonasa umbellus)
Source: BMC Res Notes. 2019 Sep 11;12:570. doi: 10.1186/s13104-019-4607-3 (PMC6737704; doi:10.1186/s13104-019-4607-3)
Supplement: Supplementary file 1 — Additional file 1. Neighbor-joining Tree of Haplotypes. This is a phenogram based on p-distances and produced with a neighbor-joining analysis (10,000 bootstrap replications) that shows relationships among the 19 haplotypes. [file 13104_2019_4607_MOESM1_ESM.pptx]

## Slide 1
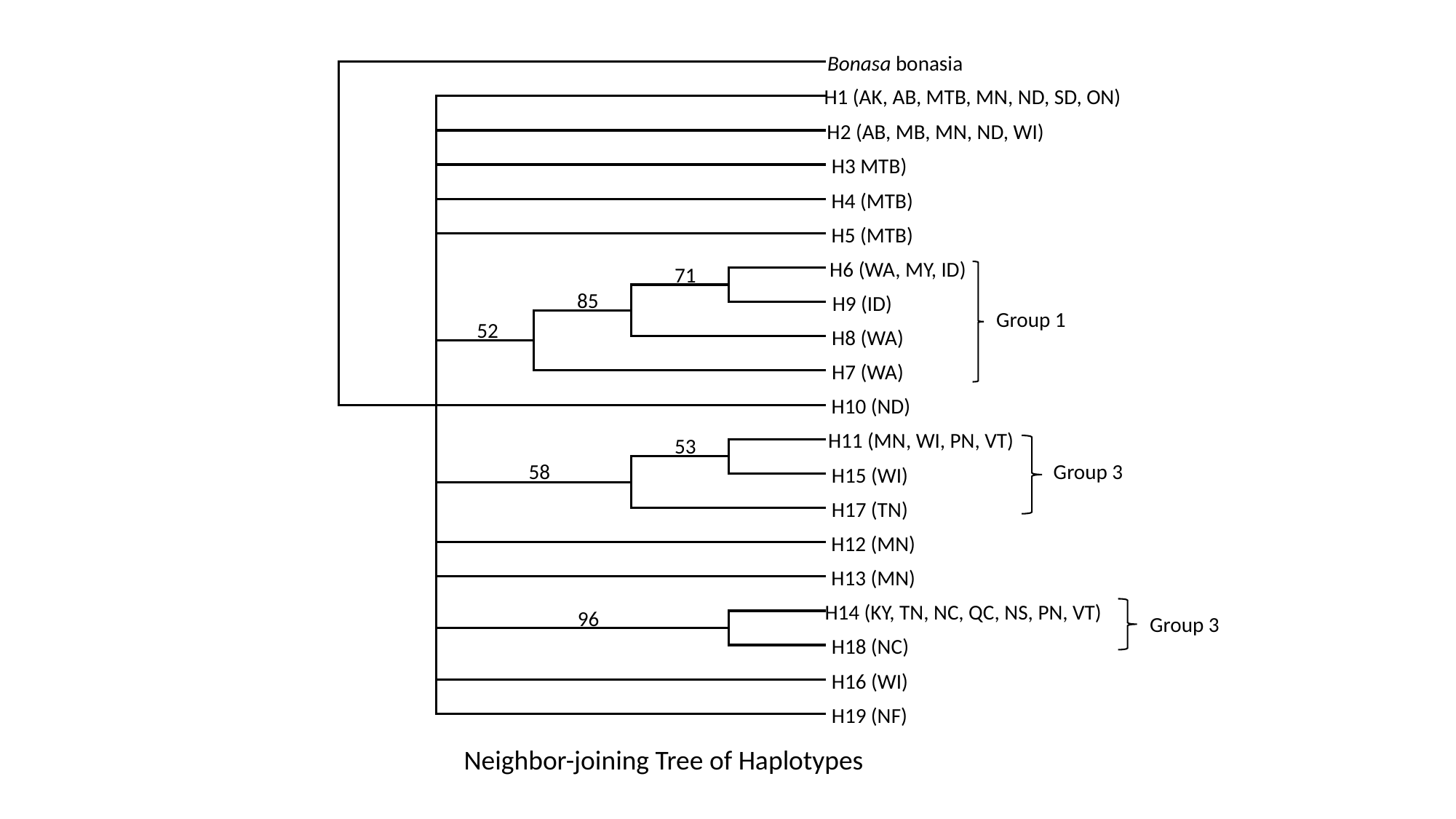

Bonasa bonasia
H1 (AK, AB, MTB, MN, ND, SD, ON)
H2 (AB, MB, MN, ND, WI)
H3 MTB)
H4 (MTB)
H5 (MTB)
H6 (WA, MY, ID)
71
85
H9 (ID)
52
H8 (WA)
H7 (WA)
H10 (ND)
H11 (MN, WI, PN, VT)
53
58
H15 (WI)
H17 (TN)
H12 (MN)
H13 (MN)
H14 (KY, TN, NC, QC, NS, PN, VT)
96
H18 (NC)
H16 (WI)
H19 (NF)
Group 1
Group 3
Group 3
Neighbor-joining Tree of Haplotypes
